# Hydrogel Bioelectronics for Health Monitoring

**DOI:** 10.3390/bios13080815

**Published:** 2023-08-14

**Authors:** Xinyan Lyu, Yan Hu, Shuai Shi, Siyuan Wang, Haowen Li, Yuheng Wang, Kun Zhou

**Affiliations:** 1 School of Science and Engineering, The Chinese University of Hong Kong, Shenzhen 518172, China; xinyanlyu1@link.cuhk.edu.cn (X.L.); siyuanwang3@link.cuhk.edu.cn (S.W.); haowenli@link.cuhk.edu.cn (H.L.); 2 The First Affiliated Hospital, Xi’an Jiaotong University, Xi’an 710061, China; 18291169116@163.com (Y.H.); karolina_tong@stu.xjtu.edu.cn (S.S.); 3 Faculty of Electrical Engineering and Computer Science, Ningbo University, Ningbo 315211, China; wangyuheng@nbu.edu.cn

**Keywords:** hydrogel, health monitoring, wearable sensor, bioelectronics, biomaterials

## Abstract

Hydrogels are considered an ideal platform for personalized healthcare due to their unique characteristics, such as their outstanding softness, appealing biocompatibility, excellent mechanical properties, etc. Owing to the high similarity between hydrogels and biological tissues, hydrogels have emerged as a promising material candidate for next generation bioelectronic interfaces. In this review, we discuss (i) the introduction of hydrogel and its traditional applications, (ii) the work principles of hydrogel in bioelectronics, (iii) the recent advances in hydrogel bioelectronics for health monitoring, and (iv) the outlook for future hydrogel bioelectronics’ development.

## 1. Introduction

The employment of wearable sensors has received great attention for its ability to monitor physiological parameters [1,2,3,4]. Most commercial wearable sensors are in the form of glasses, belts, or wristbands, which consist of rigid materials and require the additional use of bendable strips to be mounted on a human body, resulting in discomfort during the monitoring process and limiting the human physiological data obtained [5]. Therefore, to minimize the mechanical mismatch of the equipment and reduce the discomfort of users, soft wearable electronics have received tremendous attention, and they serve as a possible solution to provide user comfort with compliant mechanics [6]. Hydrogels as a three-dimensional flexible material are promising for applications of wearable bioelectronics.

**Figure 1:**
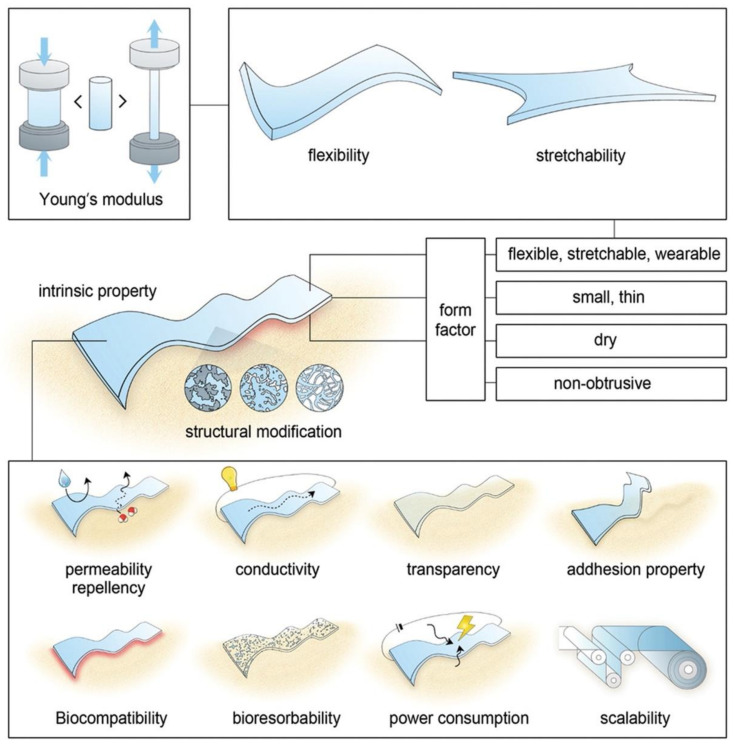
Schematic illustration of the essential material properties of wearable bioelectronics. Reproduced with permission from Wiley-VCH, copyright 2019 [8].

To further promote the developments in the field of hydrogel bioelectronics, a deep understanding of the essential properties of hydrogel bioelectronics is necessary. Figure 1 summarizes some of the essential material properties of wearable bioelectronics, based on many previous studies: the forms of wearable bioelectronics are determined by their elasticity, shapes, relative humidity, and obtrusiveness; the Young’s modulus of wearable electronics is a key factor to quantify the flexibility and stretchability of bioelectronics, while hydrogels with intrinsic flexibility and stretchability are promising materials for constructing soft wearable sensors [7,8]. The intrinsic properties of wearable bioelectronics can be directly achieved by employing hydrogels, such as transparency, adhesion, biocompatibility, and bioabsorbability: hydrogels are generally transparent in nature, and when used in electronics, the transparency of hydrogels can be advantageous for applications in visually unobtrusive devices; hydrogels exhibit excellent adhesion properties, allowing them to adhere well to various substrates, such as biological tissues, which is a crucial property to ensure the correct working location of the electronics; hydrogels are biocompatible materials, which make them suitable for constructing wearable electronic devices that contact with the skin or other biological tissues; hydrogels can be designed to be bioabsorbable, and they can be gradually broken down and assimilated by the body over time, which is a valuable property for medical devices and implants.

**Figure 2:**
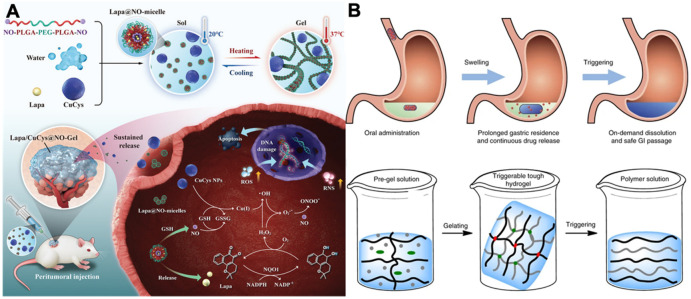
(**A**) Schematic illustration of hydrogel-based cancer therapy. Reproduced with permission from Wiley-VCH, copyright 2022 [13]. (**B**) Schematic illustration of hydrogel-based gastric resident dosage forms. Reproduced with permission from Springer Nature, copyright 2017 [14].

**Figure 3:**
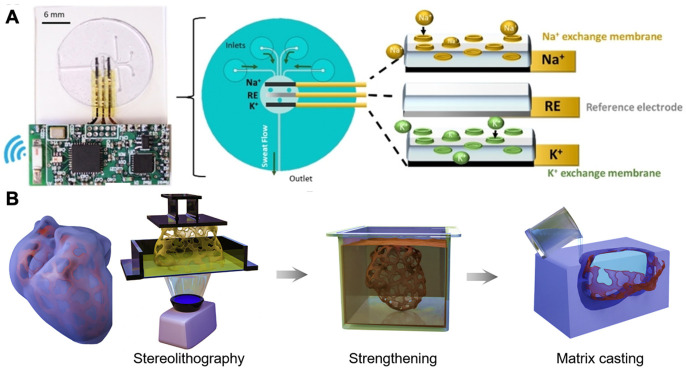
(**A**) Photograph and schematic representation of a microfluidic device integrated with hydrogel. Reproduced with permission from Wiley-VCH, copyright 2019 [17]. (**B**) Illustration of a 3D-printed hydrogel in the shape of a heart and its fabrication processes. Reproduced with permission from Elsevier, copyright 2021 [18].

Hydrogel comprises three-dimensional polymer networks that contain water [9]. As the polymer networks are infiltrated with water, hydrogels can behave like both solids and fluids and are characterized as elastic solids with softness and deformability [10,11,12]. Hydrogels have been widely employed in applications that interact with biological systems, such as therapeutic treatments and drug delivery. As shown in Figure 2A, Yu et al. designed an injectable hydrogel for synergistic tumor therapy [13], while Langer and Traverso et al. constructed a triggerable hydrogel for gastric resident dosage forms [14]. More recently, due to its superior softness, wetness, responsiveness, and biocompatibility, hydrogels have been investigated intensively for employment in sensors and electrodes to achieve various functions [15,16]. Moreover, bioelectronics based on hydrogels can perform continuous measurements of personalized biological markers from the body, and the data transmission of hydrogel bioelectronics allows doctors to obtain timely feedback on patients’ health conditions, providing a promising personalized treatment platform. For instance, Wang et al. designed a wearable hydrogel potentiometric device for the electrochemical monitoring of sodium and potassium in sweat (Figure 3A) [17], and Suo and Tang et al. employed hydrogels of heterogeneous structures to mimic biological tissues with complex shapes and high fatigue resistance as shown in Figure 3B [18]. 

To date, numerous hydrogel-based bioelectronics have been developed for health monitoring. However, there has yet to be a comprehensive summary of hydrogel-based bioelectronics for health monitoring. Thus, this review first introduces hydrogel and its traditional applications, and then the work principles of hydrogel-based bioelectronics are discussed. Furthermore, the recent advances in hydrogel-based bioelectronics for health monitoring are presented. The contents discussed under this topic mainly include hydrogel for health data analysis, wound dressing, inflammation treatments, oral care, and some nerve-related applications. We hope this review provides an overview of hydrogel-based bioelectronics for health monitoring and stimulates more interesting ideas in this field.

## 2. Principles of Hydrogel

Hydrogel comprises polymer networks infiltrated by water, with water absorption and moisture retention properties [19]. Hydrogels are formed by cross-linking polymer molecular chains dispersed in an aqueous medium through a variety of mechanisms, including physical entanglement, chemical bonding, van der Waals forces, etc. Due to a porous three-dimensional structure, excellent stretchability, and intrinsic biocompatibility, flexible bioelectronics concentrated on conductive hydrogels have attracted much attention, and hydrogels can be employed as a part of a sensor to measure and monitor physiological indicators of the human body. The elasticity, electrical conductivity, dielectric properties, and adhesion properties of hydrogels can be monitored by the molecular chain length, degree of crosslinking, the crosslinking mechanism, the type of monomer functional group, and the water content of the hydrogel [20]. 

Conductive hydrogels are commonly prepared by integrating conductive components or polymers into a hydrogel matrix with an extensive range of modulation of network structure to achieve different electrochemical properties, mechanical properties, and biological functions [21]. More importantly, by changing the conductive filler, dopant, crosslinking or hydration state, the unique properties and versatility of conductive hydrogels can be applied to various promising fields, such as wearable sensors, human–machine interfaces, etc. [22]. A straightforward method to prepare conductive hydrogels is the direct doping of hydrogels with soluble inorganic salts as the high-water content and microporous structure of the hydrogel can facilitate the dispersion of salts to give the hydrogel ionic conductivity, which is known as physical doping [23]. Another type of doping is chemical doping, where ions act as ligand centers to crosslink different polymer chains in the hydrogel to form a hydrogel network, resulting in ionic conductor-based conductive hydrogels that have unique properties of hydrogel such as adhesion, self-healing, and biocompatibility [24]. Moreover, liquid metals (LMs) can also be applied in hydrogels to form conductive hydrogels because LMs possess high thermal conductivity, good electrical conductivity, flexibility, low toxicity, and ductility [25]. Compared with rigid conductive materials, LMs can match well with stretchable substrates, thus avoiding internal stress concentration and forming ductile composites. Although metal ions can provide good ionic conductivity for hydrogel bioelectronics, they may cause undesirable electrochemical reactions at the contact interface, which is detrimental to the long-term stable operation of the sensors. Therefore, the doping of hydrogels with highly conductive nanomaterials rather than metal ions has been recognized as one of the most common strategies to prepare conductive hydrogels [26]. Based on the chemical composition, these conductive nanofillers can be classified into carbon-based nanomaterials and metal-based nanomaterials. Furthermore, conductive polymers, which are based on long-chain polymers consisting of carbon atoms and a conjugated π-electron system, are another promising way to prepare conductive hydrogel. Conductive hydrogels have many advantages over other conductors, including better processability and conductivity than ionic conductors [24,27,28]. In recent years, various conductive polymers with different conductivity properties have been reported, such as poly(3,4-ethylenedioxythiophene):polystyrene sulfonate (PEDOT: PSS), polyaniline (PANI), and polypyrrole (PPy) [29,30,31]. The comparison of the ensile strength and conductivity between conductive hydrogels and metal oxide semiconductors described in the literature was conducted (Table 1): hydrogel in general has considerably more tensile strength and conductivity than metal oxide semiconductors; hydrogels also exhibit lower values of the Young’s modulus than metal oxide semiconductors, according to the literature, which means hydrogels have better elasticity; in addition, conductive hydrogels exhibit excellent sensitivity to a variety of external signals. Compared with other flexible materials, hydrogels are favorable for constructing bioelectronics due to their intrinsic flexibility, conformability, biocompatibility, transparency, high water content, etc. However, the swelling and dehydration of hydrogels may limit their applications, and the long-term stability of hydrogel bioelectronics is another challenging issue.

According to the differences in conductive fillers, conductive hydrogels can be divided into ionic hydrogels and electronic hydrogels.

### 2.1. Principles of Electronic Conductive Hydrogels

Electronic conductive hydrogels are fabricated via chemically bonded, ionically crosslinked, and self-assembled conductive fillers, such as carbon nanotubes, graphene, and metal nanoparticles, to build three-dimensional electron-transporting conductive networks.

**Figure 4:**
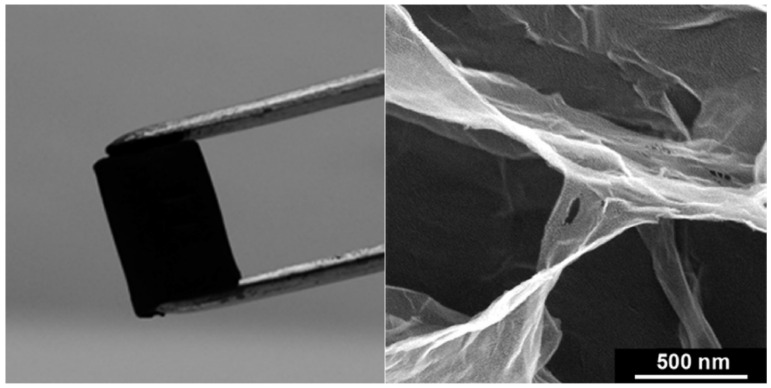
Images of self-assemble graphene hydrogel. Reproduced with permission from the American Chemical Society, copyright 2010 [38].

Graphene is a commonly used filler for electronic conductive hydrogels due to its flexible two-dimensional structure and excellent electronic properties. Graphene nanosheets can be agglomerated in aqueous solution to form physically interconnected three-dimensional porous networks, and the resulting graphene hydrogels, as shown in Figure 4, contain about 2.6 wt% of graphene and 97.4 wt% of water, showing electrical conductivities of up to 0.5 S m^−1^ [38]. 

Alternatively, conductive polymers can self-assemble to form hydrogels. PANI, an intrinsically insulating polymer, can construct electronic conductive hydrogels via protonation. For example, it can be crosslinked with phytic acid to form conductive hydrogels with a high conductivity of 11 S m^−1^ [39]. In addition, PEDOT can be directly crosslinked to form hydrogels with very high conductivity. Pure PEDOT:PSS hydrogels were prepared by adding the nonvolatile solvent dimethyl sulfoxide (DMSO) to an aqueous solution of PEDOT:PSS, annealed by drying in a controlled manner, and then reswollen in water to give pure PEDOT:PSS hydrogels with a conductivity of 2000 S cm^−1^ in phosphate-buffered saline and 4000 S cm^−1^ in water [29]. The Young’s modulus of this conductive hydrogel was 2 MPa, and the tensile strength was more than 35%.

### 2.2. Principles of Ionic Conductive Hydrogels

A large amount of water and electrolytes can serve as an ion reservoir in the human body. Ions are the main way to transmit signals in human body, and bioelectronic activities are closely related to the ionization and ion migration that occurs in biological systems. In the practical application of flexible bioelectronics, the match between their mechanical properties and the tissue of the organism is a crucial index. If there is a large difference between the mechanical properties of the flexible bioelectronics and the organism, direct physical damage to the organism may result in and significantly reduce the working efficiency of the device.

**Figure 5:**
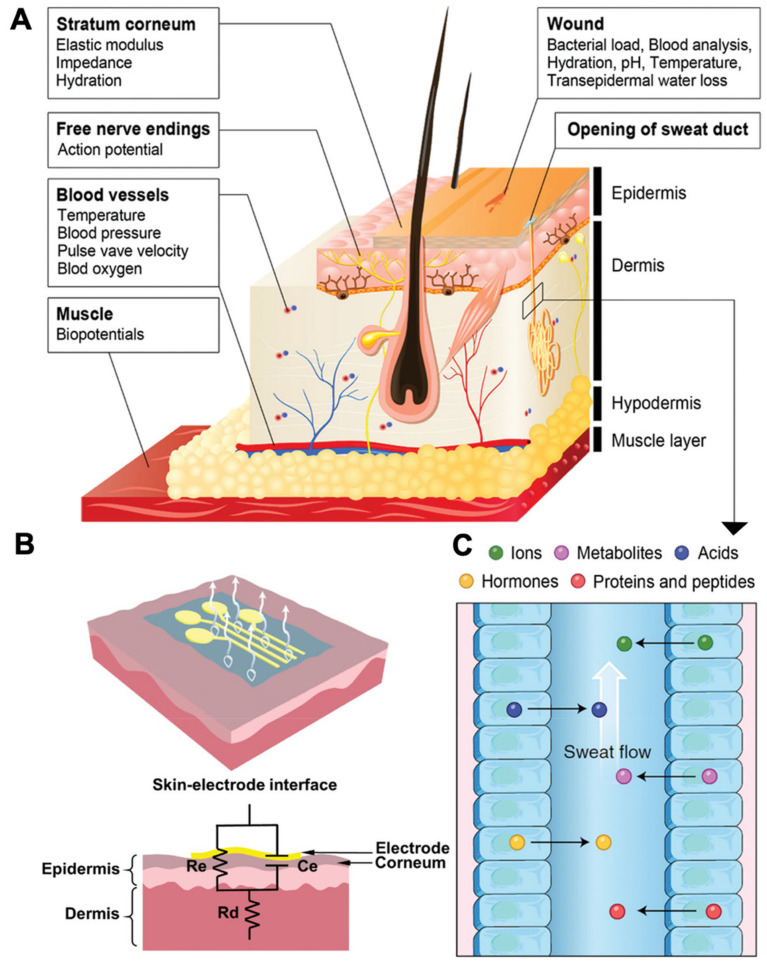
(**A**) Overview of a skin-based sensing platform. Reproduced with permission from Wiley-VCH, copyright 2019 [8]. (**B**) Schematic illustration of a skin–electrode interface model of on-skin electrodes. Reproduced with permission from Wiley-VCH, copyright 2021 [43]. (**C**) Schematic illustration of various analytes in sweat from (**A**). Reproduced with permission from Wiley-VCH, copyright 2019 [8].

To achieve the real-time visualization of data transmission, hydrogel-based bioelectronics are constructed based on various types of sensors, such as temperature sensors, pH sensors, and hydration sensors, which can be embedded in hydrogel or contacted with hydrogel surface to acquire related information and data. As shown in Figure 5A, human skin, the largest organ of the integumentary system of the human body, offers various crucial biological signals from the epidermis, inner organs, muscles, etc. [8]. For instance, there are multiple analytes, including hormones, proteins, and peptides, can be obtained from the sweat. Since the body may move continuously, the conformal contact of hydrogel bioelectronics with the monitoring site allows for more accurate detection. Moreover, while wet electrodes are unsuitable for long-term detection due to potential drying and skin irritation issues, dry electrodes can achieve long-term noninvasive monitoring, as moisture on the skin can enhance the stabilization of the dry electrodes [40,41,42]. The skin–electrode interface and circuit model of an on-skin electrical sensor is shown in Figure 5B based on a parallel circuit of leakage resistance and capacitance to construct a conformable electrode–skin interface [43]. The signal acquired by the hydrogel sensors can be transmitted to external devices via wires or wireless transmission. As the external device receives signals from the hydrogel bioelectronics, signal processing and data analysis are performed, which allows users to visualize their physiological parameters and is useful for assessment of health status, providing a promising platform for personalized healthcare. 

**Figure 6:**
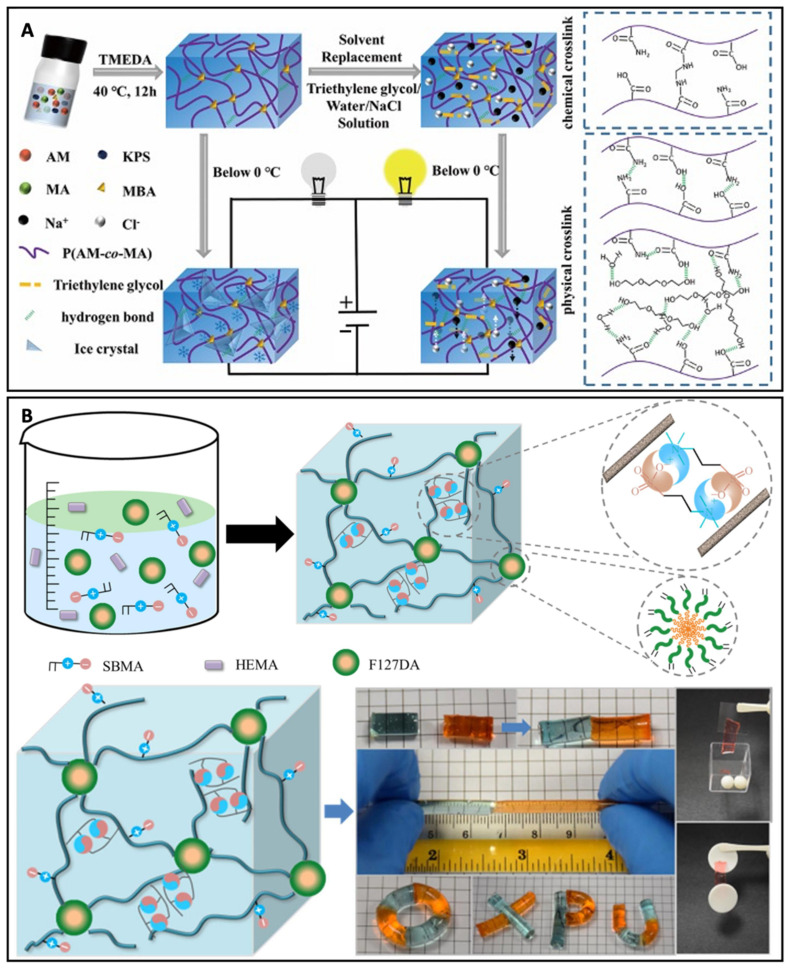
(**A**) Schematic illustration of ionic conductive hydrogels’ formation, structure, and intermolecular interactions. Reproduced with permission from Wiley-VCH, copyright 2022 [44]. (**B**) Schematic illustration of the fabrication process of the nano-micelle zwitterionic hydrogels. Reproduced with permission from Elsevier, copyright 2020 [45].

Wang, Lai, Li, and Wu et al. reported a ionic conductive hydrogel (Figure 6A), which was a dual-crosslinked organohydrogel fabricated by immersing the poly(acrylamide-co-maleicacid) hydrogels in triethylene glycol/sodium chloride/water solution with electrical conductivity and excellent stretchability (1322 ± 75%), even at low temperature [44]. Sun and He et al. reported a ionic zwitterionic nano-micelle hydrogel, which was fabricated via in situ co-polymerization of zwitterionic monomer SBMA and nonionic monomer-hydroxyethyl methacrylate (HEMA) using Pluronic F127 di-acrylate (F127DA) as macro-crosslinkers (Figure 6B) [45]. The ionic conductive hydrogels could sustain a strain of 98% without fracture and featured a tensile fracture strain of about 1000% with excellent adhesiveness and conductive properties.

## 3. Recent Advances in Hydrogel Bioelectronics for Health Monitoring

Hydrogel is regarded as a promising candidate for bioelectronics with its distinctive advantages of pleasant flexibility, various functionality, favorable biocompatibility, and excellent sensing properties. Hydrogel bioelectronics can detect biophysical signals, like body temperature and human activity, biochemical signals, as in sweat, and so on, forming a compatible sensor for long-term continuous health monitoring. The recent progress of hydrogel bioelectronics can be categorized into by its functions: (1) health data analysis, (2) wound dressing, (3) inflammation treatments, (4) oral care, and (5) some nerve-related applications.

### 3.1. Hydrogel Bioelectronics for Health Data Analysis

Hydrogel bioelectronics can be used to detect various human physiological metabolites and health-related parameters, such as sweat, blood, human activities, etc. Continuous real-time monitoring of individuals’ health data can be achieved, which enables doctors to obtain timely feedback on patients’ health conditions, providing a promising personalized treatment platform.

**Figure 7:**
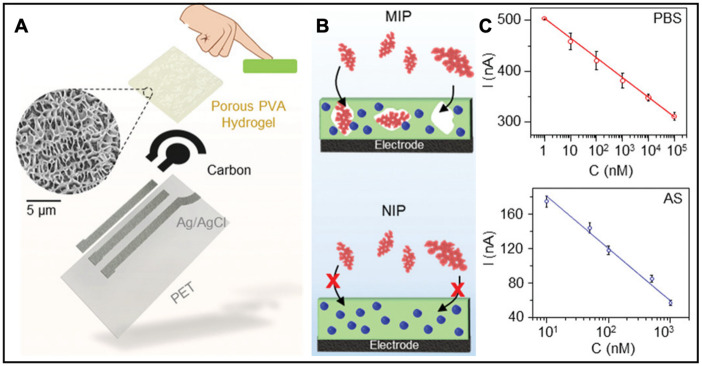
(**A**) Description of the touch-based fingertip cortisol sensor. (**B**) Schematic interaction of cortisol in molecularly imprinted polymers (MIP) and lack of interaction in non-imprinted polymer (NIP). (**C**) Calibration curve of the electrochemical response of the MIP sensor to different cortisol concentrations in phosphate buffer solution (PBS) and in artificial sweat (AS). Reproduced with permission from Wiley-VCH, copyright 2021 [48].

The noninvasiveness of bioelectronics is crucial for developing safe, effective, and widely applicable health data analysis. Noninvasive bioelectronics enable continuous real-time tracking of health indicators without causing significant harm or discomfort to the human body, making them more accessible to the public. Epidermal sweat sensing, as one of the noninvasive ways to detect physiological signals, contains various essential biological parameters, such as ions, small molecules, and macromolecules [46]. Hydrogel with its distinctive biocompatibility and flexibility advantages can incorporate epidermal sweat sensing and electrochemical technology to construct a continuous health data visualization platform. Wang et al. developed an epidermal sweat-sensing platform to detect and directly display the concentration of health-related parameters in sweat [47]. The sweat-sensing platform was integrated with the electrochemical sensors, a battery, hydrogel, and an electrochromic display. The high-performance battery was characterized by its flexibility. At the same time, the polyvinyl alcohol (PVA) hydrogel was employed as an electrolyte interface to contain the electrolyte and avoid any leakage, which enabled the sweat-sensing platform to be used in practical applications. The integrated hydrogel bioelectronics were robust to mechanical deformation, as they were unaffected by 1500 stretching cycles at 20% strain. The real-time data visualization was achieved by the electrochromic display (PEDOT:PSS): the electrochromic display was fabricated by a top PEDOT:PSS panel and ten separately addressable pixels with highly viscous ionic pathways between the two panels and to avoid short-circuiting and corrosion. The PEDOT:PSS demonstrated good stability, as the electrical current response only showed a slight decrease and high reproducibility in the peak current after 10,000 on/off cycles. The detection of Na^+^ ions in sweat was illustrated by the color change of the PEDOT:PSS panel: when the PEDOT:PSS was reduced by electrons, the color changed from light blue to dark blue, achieving visualization of the pH of analytes. In addition to monitoring the sodium ions and pH of sweat, hydrogel bioelectronics can also detect other physiological parameters in sweat, like cortisol, which plays a crucial role in illustrating human stress levels. Wang et al. constructed a highly selective touch-based hydrogel platform for sensing the cortisol level [48]. The highly permeable PVA hydrogel collected sweat rapidly and effectively from the fingertip (Figure 7A). The molecularly imprinted polymer (MIP) detection depended on the selective binding of cortisol to the imprinted PPy membrane, which was synthesized PPy with cortisol as the template, to achieve effective cortisol sensing. Compared with a non-imprinted polymer (NIP), an MIP with a porous structure allowed cortisol to occupy the spacing and hinder the charge transfer of the embedded redox probe as a signal for the cortisol level (Figure 7B). The sensor can monitor a wide range of cortisol concentrations in PBS (Figure 7C). Furthermore, the data generated in artificial sweat (AS) showed great reproducibility compared with that in PBS, demonstrating remarkable sensitivity in cortisol sensing (Figure 5E). Therefore, a real-time cortisol detection platform was constructed to monitor the cortisol level in fingertip sweat using the porous hydrogel and the PPy-based MIP. 

**Figure 8:**
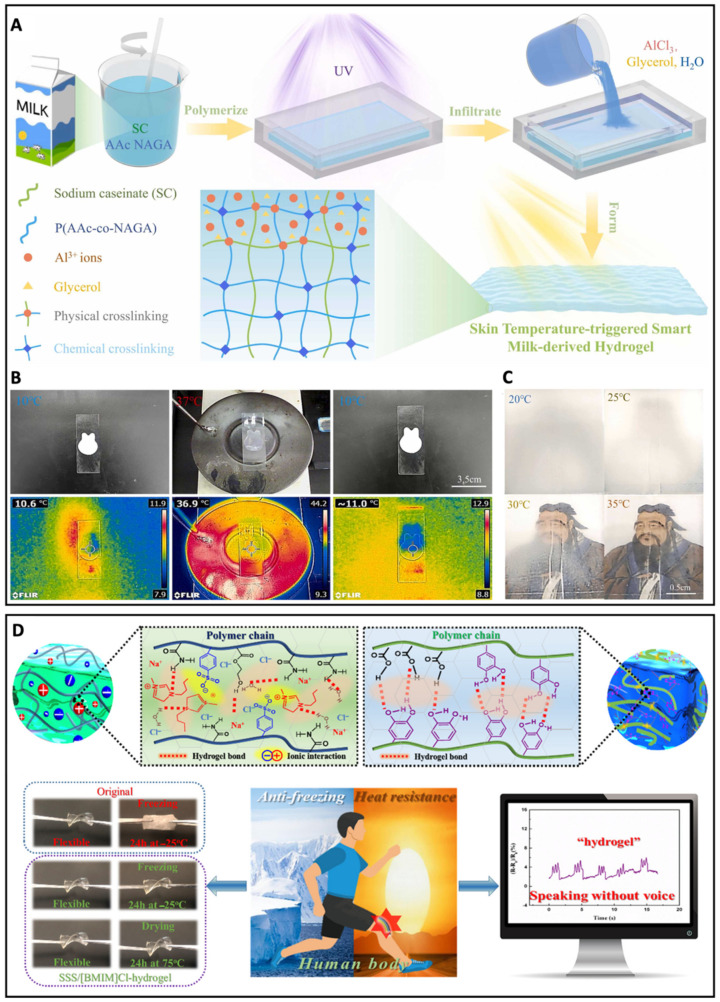
(**A**) Schematic representation of the preparation of the skin-temperature-triggered smart milk-derived hydrogel (STSMH). (**B**) Infrared thermal images of the phase transition process of the STSMH hydrogel. (**C**) Visual presentation of the invisible cloak. Reproduced with permission from Elsevier, copyright 2022 [55]. (**D**) Illustration of multiple bonding interactions in the double-layer hydrogel and its ability to detect human motion. Reproduced with permission from the American Chemical Society, copyright 2022 [58].

In addition to detecting physiological parameters, hydrogel bioelectronics can also be used to monitor human motions, temperature management, and so on, which is promising for personalized healthcare development. Compared with common hydrogels, ionic hydrogels are favorable for developing wearable intelligent bioelectronics with its unique advantages of rapid water absorption and significant water retention properties [7,49,50,51]. To satisfy the demand for smart sensing, hydrogels are usually fabricated by adhesive constituents, like dopamine [52]. However, single-targeted adhesive hydrogels have limited adhesion functionality and insufficient stability, which can only adhere to the target substrate and are difficult to reposition on the material [53,54]. To address these challenges and facilitate the use of wearable bioelectronics based on ionic hydrogel, it is crucial to develop smart adhesive hydrogels that can repeatedly attach and detach as needed. Jin et al. solved this issue by creating a skin temperature-triggered smart milk-derived hydrogel (STSMH) [55]. The adhesion/non-adhesion behavior of STSMH can be monitored by temperature change as a stimuli-response, which was achieved by the thermosensitive hydrogel: the milk-derived sodium caseinate (SC) in the upper critical solution temperature (UCST)-typed thermoresponsive polymer was infiltrated by polyvalent metal ions one-sidedly, resulting in the switchable adhesive hydrogel for on-demand detachments (Figure 8A). On the other hand, the STSMH demonstrated significant color-changing abilities, which can be used for visually monitoring the temperature, multilevel temperature-dependent encryption, and wearable bioelectronics. As shown in Figure 8B, the STSMH hydrogel was opaque at room temperature and changed to transparent when the temperature reached 37 °C, the human body temperature. The STSMH hydrogel can sensitively perceive external strain and temperature variation by electrical signals. The temperature sensitivity of the STSMH hydrogels was quantified by the temperature coefficient of resistance (TCR), and the TCR was −4.03%/°C in the room temperature range, while beyond the range of the human body temperature, the TCR decreased to −0.74%/°C, indicating excellent distinction between the external and human body temperature. The thermoresponsive property of the STSMH was beneficial for visual perception. Specifically, the gauge factor (GF) of the STSMH hydrogels was 3.17 in the strain range of 0–50% and dramatically increased to 37.59 in the strain range of 275–300%, demonstrating the outstanding sensitivity of detecting tensile strain. The STSMH hydrogels also exhibited a rapid correspondence time (169 ms) and short recovery time (250 ms). The transparency-switching process can also be used in encryption as shown in Figure 8C: the portrait of Confucius was invisible at room temperature, but as the temperature increased, the portrait appeared gradually, which was promising for applications in concealment and preservations of valuable objects. However, some hydrogels cannot be used in extreme environments and have low adhesion, resulting in difficulties for hydrogel-based wearable electronics to apply for monitoring motions [56,57]. Zhang and Zhang et al. developed an anti-freezing and nondrying hydrogel sensor for human motion detection [58]. The hydrogel was constructed with a double layer configuration (Figure 8D): the top-layer hydrogel could be used for sensing and conducting, while the bottom-layer hydrogel demonstrated an excellent adhesion property. Intermolecular hydrogen bonds and ionic interactions achieved the conductivity of the top-layer hydrogel. Moreover, one ionic liquid was employed to construct the top-layer hydrogel, which contributed to anti-freezing and non-drying properties and allowed the bilayer hydrogel to be used for motion monitoring in harsh environments.

### 3.2. Hydrogel Bioelectronics for Wound Dressing

Skin wounds, which are caused by the destruction of the skin integrity, cannot be healed immediately [59]. Inadequate care for wounds can cause severe infections and might lead to serious aftermath, such as tissue damage, disability, and septicemia [60,61,62]. Therefore, wound dressing is crucial for repairing the skin and restoring cutaneous functions. However, conventional dressing, like bandages, requires frequent replacements [63], increasing the risk of infection and the possibility of secondary injury. Hydrogel, a recently developed material, can be used to solve the issue mentioned above, because hydrogel can effectively create a physical barrier against bacterial infection, avoid secondary injury, and so on [64]. Moreover, hydrogel with adjustable functionality can also achieve on-time monitoring and medical treatment of the wound.

**Figure 9:**
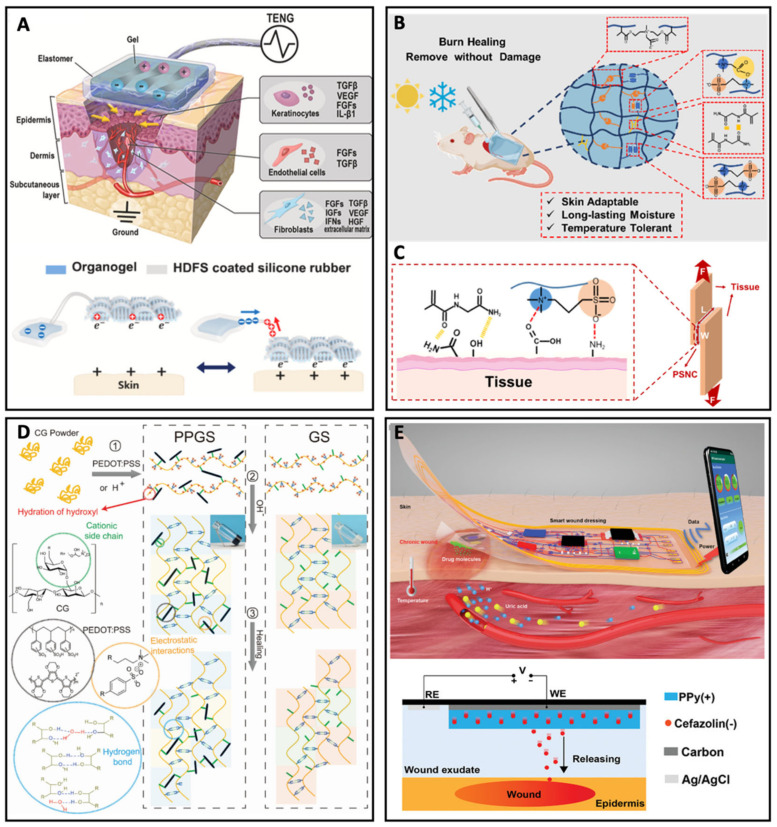
(**A**) Schematic illustration of the wound dressing mechanism of the iTENG patch and its biomechanical energy harvesting mechanism. Reproduced with permission from Elsevier, copyright 2020 [65] (**B**) Schematic illustration of the hydrogel dressings for burn wound healing. (**C**) Schematic illustration of the adhesive mechanism of PSNC hydrogels. Reproduced with permission from the American Chemical Society, copyright 2021 [66]. (**D**) Schematic illustration of the fabrication and self-healing of the hydrogel. The red, green, black, orange, and blue dashed lines represent the hydration of the hydroxyl, cationic side chain, PEDOT:PSS, electrostatic interactions, and hydrogen bond, respectively. Reproduced with permission from Wiley-VCH, copyright 2020 [67]. (**E**) Schematic illustration and side view of a battery-free and wireless wound dressing for monitoring wound infection and electric-controlled drug delivery, and the schematic illustration of the electrically controlled drug delivery system. Reproduced with permission from Wiley-VCH, copyright 2021 [68].

Sun et al. designed a hydrogel-based ionic patch for accelerated wound healing [65]. The ionic triboelectric nanogenerator (iTENG) patch was composed of a hydrogel platform, a TENG, a wire, and a patch for harvesting biomechanical energy and transferring electric potential to an injured tissue. The patch was directly used on the cutaneous wound, and it accelerated the wound healing process due to the endogenous electric field (EF) effect generated by the exogenous EF transfer from real-time biophysical energy harvesting. As shown in Figure 9A, the iTENG can induce a temporary electric potential difference between the patch and the wound, which allows charged ions to move through the ion channels in the epidermis, and induces the secretion of biomolecules, such as transforming growth factor (TGF), vascular endothelial growth factor (VEGF), fibroblast growth factor (FGF), and epidermal growth factor (EGF). The ion channels in the epidermis were connected to the hydrogel-based patch for ionic integration and maximization of contact electrification from the friction between the wound tissue and the patch surface. On the other hand, a burn wound can be considered the most challenging and severe type of wound. Feng, Wang, and Dong et al. reported a dual crosslinked hydrogel, named PSNC, for burn injury dressing with skin adaptability, long-lasting moisture, and temperature tolerance (Figure 9B) [66]. The PSNC hydrogel was fabricated with polymerized sulfobetaine methacrylate, N-(2-amino-2-oxyethyl)acrylamide (NAGA), and 1-carboxy-N-methyl-N-di(2-methacryloyloxy-ethyl)methanaminium inner salt to combine with the hydrogen bonding of NAGA with a covalently cross-inked zwitterionic network, which endowed the hydrogel with skin-like mechanical properties. The skin-like mechanical properties were quantified by the high stretchability of 1613.8 ± 79.8%, a tensile strength of 77.5 ± 1.8 kPa, and a tensile modulus of 1.9 ± 0.1 kPa. Lap-shear tests were performed to test the adhesive capacity of the PSNC hydrogels (Figure 9C): the PSNC hydrogel, compared with the commercial fibrin sealant, had stable adhesion, a similar lap-shear strength, and adequate burst pressure.

Wound healing on stretchable parts of the human body, such as elbows, knees, and ankles, is much more difficult due to their frequent movements and requires dressing with superior compliance and self-healing ability to avoid secondary damage and shorten the healing time [69,70,71]. Self-healing hydrogel has drawn great attention, but the complicated preparation processes are a large challenge. Zheng, Yang, and Jiang et al. constructed a self-healing hydrogel, named poly(3,4-ethylenedioxythiophene): poly(styrenesulfonate)/guar slime (PPGS), with a rapid fabrication process [67]. Guar gum, a water-soluble galactomannan derived from soybean gum, was employed to form the self-healing hydrogel by adding a small amount of acid to speed up the hydration rate of the cationic guar gum (CG), and the following neutralization changed the CG to a hydrogel state (gaur slime, GS) at physiological pH (Figure 9D). The fabrication process can be finished within 1 min. Furthermore, a conductive solution was used to form PPGS for improving the conductivity and healing capacity in wound closure and tissue reorganization.

A smart wound platform that monitors wound status and provides timely therapies is promising for clinical use and personalized healthcare. If the smart platform for wound dressing could achieve controlled drug delivery, the quality of the therapeutic effects would increase significantly. Liu et al. recently developed a wireless and battery-free hydrogel platform with continuous point-of-care of the wound and on-demand drug delivery [68]. The smart dressing was constructed of two layers: the top layer was a flexible circuit board for transmitting wireless data, monitoring temperature, and processing on-site signals, while the bottom layer was composed of various stretchable electrodes, including a uric acid sensor, a pH sensor, and a drug delivery electrode (Figure 7D). Compared with thermo-responsive materials that might lead to burns and lessen the wearing comfort, an electric-controlled drug release module was integrated into the smart dressing. When bacteria infected the wound, the hydrogel dressing would monitor changes in the level of uric acid, pH, and temperature, and the information would be transmitted wirelessly to a smartphone to evaluate whether therapy was needed. 

### 3.3. Hydrogel Bioelectronics for Inflammation Treatments

Inflammation is strongly associated with infection and the defense mechanism in the immune system, which can cause injury and affect the body’s ability to repair [72,73]. Drug therapy is usually used to treat inflammatory conditions, and biomaterials can be used as drug carriers to achieve controlled delivery with high efficacy and few side effects. Hydrogel bioelectronics have attracted significant attention in inflammation treatment: on the one hand, drug-loaded treatment can be achieved by hydrogel due to its high loading capacity and stimuli-responsive properties; on the other hand, it can transmit signals to construct a theranostic platform [74,75]. Hydrogel is a promising material for achieving inflammation treatments and continuous management in wearable health monitoring devices. 

**Figure 10:**
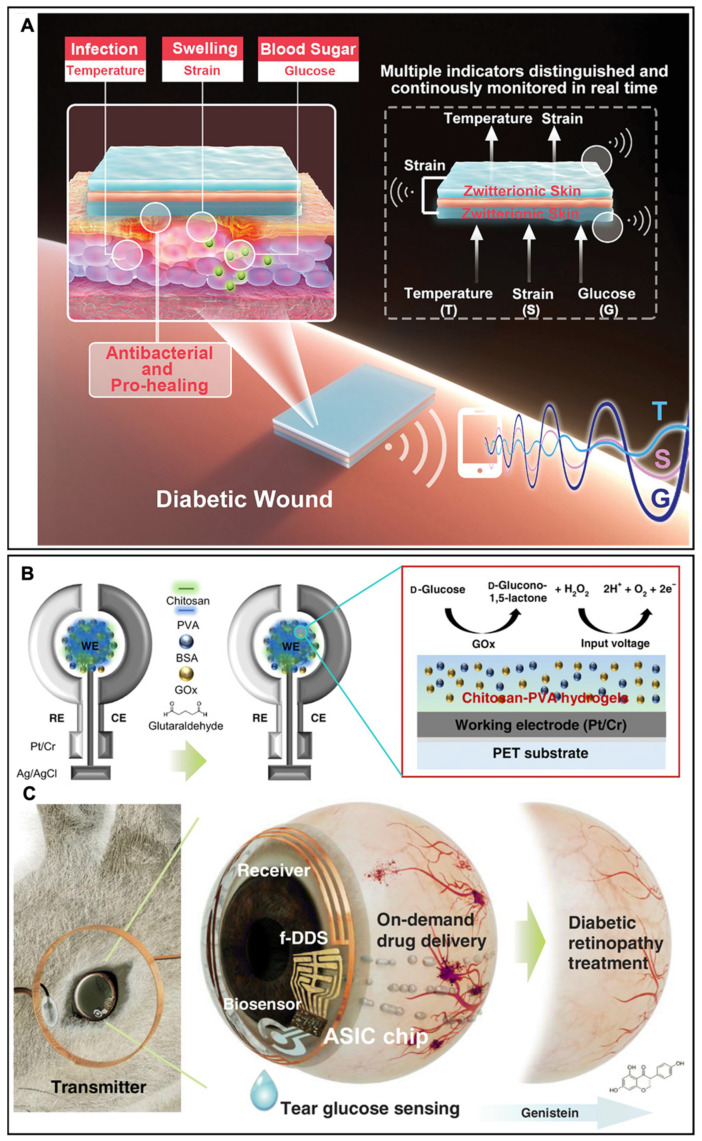
(**A**) Schematic illustration of the hydrogel skin-like sensor system for a diabetic inflammation wound. Reproduced with permission from Wiley-VCH, copyright 2021 [78]. (**B**) Schematic illustration of the hydrogel-based ocular glucose sensor. (**C**) Schematic illustration of a smart contact lens for diabetic diagnosis and therapy. Reproduced with permission from the American Association for the Advancement of Science, copyright 2020 [79].

Diabetic ulcers are chronic wounds that are difficult to heal. They are easily inflamed due to impaired immune functions and hyperglycemia, which can cause the constriction of blood vessels and rigid cell membranes [76,77]. To efficaciously monitor the diabetic wound microenvironment, Yang and Zhang et al. designed a thermo-glucose-responsive skin-like hydrogel to monitor and distinguish multiple signals, including temperature, strain, and glucose concentration for establishing the platform of continuous wound management and treatment for diabetic patients [78]. The hydrogel (SB-N-MB) was incorporated with zwitterionic carboxyl betaine (SBMA), N-isopropyl acrylamide (NIPAAm), and methylacrylamide phenylboric acid (MPBA) to endow the SB-N-MB hydrogel’s properties of temperature-response, glucose-response, and strain-response (Figure 10A). The SB-N-MB hydrogel demonstrated excellent elasticity, as when the hydrogel was compressed to 50% of its original height, it could recover to 100%. In addition, the tensile strain of the SB-N-MB hydrogels was 273%, and the tensile stress reached 70 kPa, which was seven times larger than the comparison group, indicating the SB-N-MB hydrogels possessed excellent stretchability and were favorable for inflammation treatments. The diabetic wound platform was further constructed based on a sandwich structure sensor: the upper and lower layers were based on the SB-N-MB hydrogel to monitor the parameters of temperature, strain, and glucose, while the middle isolation layer was VHB elastomer. Therefore, the resulting sandwich SB-N-MB hydrogel-based platform achieved continuous real-time monitoring of the wound microenvironment, distinguishing three signals, and proactive healing of the diabetic wound.

Aside from diabetic wounds, diabetes can cause physiological abnormalities and also lead to some severe inflammatory complications, like neuropathy [80] and diabetic retinopathy [81]. Diabetic retinopathy, one main cause of blindness, can cause the loss of vision as some inflammatory molecules will increase their expression in the diabetic milieu and then propagate inflammation in the retina, impairing the functions of the eyes [82]. Therefore, anti-inflammatory treatments of diabetic retinopathy promise to address the vision loss issue caused by diabetes. Moreover, the cornea surface uniquely provides a noninvasive interface to access physiological parameters in the human body, as the eyes are directly connected to multiple important organs, such as the brain, heart, and lungs [83,84]. Therefore, developing a system, which can achieve anti-inflammatory treatments and the real-time detection of parameters related to diabetic retinopathy, will significantly enhance the theranostic efficacy of diabetic retinopathy. Hahn et al. developed a wireless hydrogel contact lens for the remote monitoring of diabetic diagnosis and diabetic retinopathy therapy [79]. Glucose oxidase (Gox), bovine serum albumin (BSA), and chitosan were fabricated on the hydrogel electrode to monitor the glucose content in tears with improved sensitivity and stability (Figure 10B). The contact lens can monitor the glucose concentration in tears noninvasively instead of invasive blood tests, while the working electrode allowed wireless powering from an external power source with a transmitter coil, enabling on-demand drug delivery of genistein and remote communications for diabetic diagnosis and diabetic retinopathy therapy (Figure 10C). Therefore, hydrogel with adjustable functionalities can be employed as a favorable platform to detect inflammation-related parameters and achieve therapeutic treatments simultaneously.

### 3.4. Hydrogel Bioelectronics for Oral Cares

Monitoring the health of the oral cavity is crucial and essential as the oral cavity includes some important organs, like teeth, and diseases in the oral cavity might lead to discomfort and serious complications, leading to major impacts on the quality of life [85]. Therefore, it is urgent to develop bioelectronics for oral cavity monitoring. Hydrogel dressing attracts great attention due to its unique structure and integration capacity. However, most hydrogel dressings are applied to dry skin wounds, and to employ hydrogel in the oral cavity, hydrogel with excellent adhesion and minimized swelling in a moist environment is needed [86,87].

**Figure 11:**
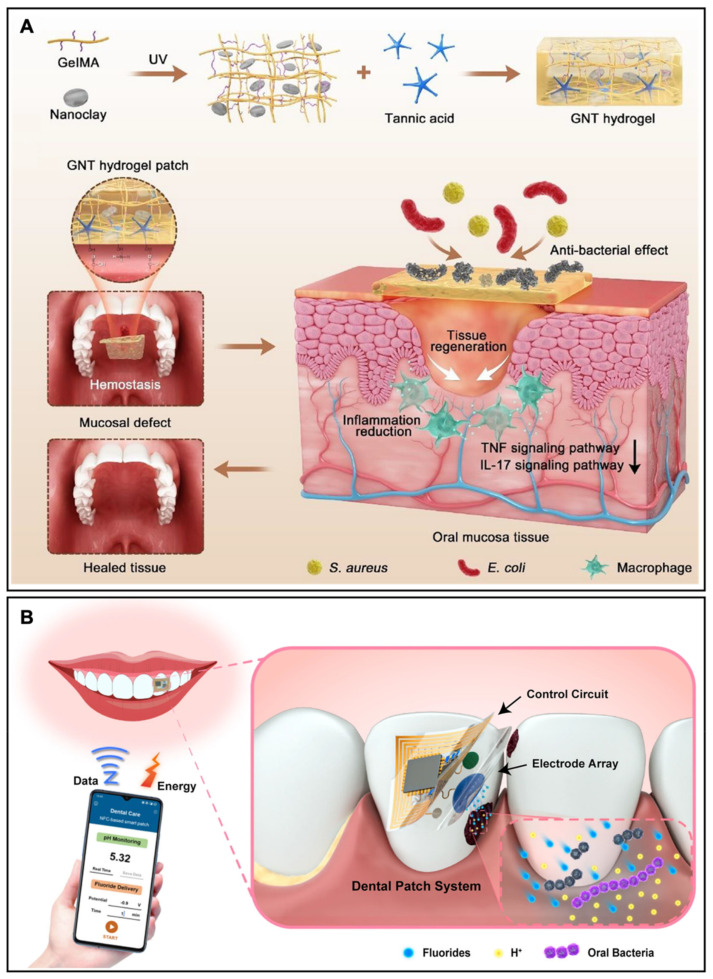
(**A**) Schematic illustration of an antibacterial oral GNT hydrogel. Reproduced with permission from the American Chemical Society, copyright 2022 [88]. (**B**) Schematic illustration of the hydrogel patch for on-demand fluoride delivery and real-time monitoring of the tooth microenvironment. Reproduced with permission from Springer Nature, copyright 2022 [89].

Chen and Wang et al. reported a low-swelling GNT hydrogel for curing a full-thickness oral mucosal defect with rapid hemostasis and anti-inflammation [88]. As shown in Figure 11A, a GNT hydrogel was designed according to a dual cross-linking strategy and an environmentally friendly fabrication, which consisted of gelatin methacrylate (GelMA), nanoclay, and tannic acid (TA), resulting in a minimized swelling adhesive hydrogel that was suitable to improve the stretchability and use in oral mucosal care. GNT hydrogels were characterized by not only low swelling but also good stretchability (211.86%). GNT hydrogel exhibited a stable appearance and adhered to the oral mucosa without detaching for over 10 h, while the GelMA deformed and degraded in the oral environment. The adhesive strength of the GNT hydrogels was 5 times larger than that of GelMA, demonstrating its significant adhesive properties. Contrary to conventional therapy, which separated hemostasis and anti-inflammation, GNT hydrogel was able to achieve both simultaneously: the RNA sequencing analysis indicated the GNT hydrogel reduced inflammation levels by downregulating multiple inflammation-related pathways, advancing the therapeutic effects of GNT hydrogel.

Dental diseases are associated with bacterial infection and the inflammatory response of the dental tissues, which can lead to discomfort and the destructive infection of tooth-supporting tissues [90,91]. One specific dental disease, periodontitis can cause periodontal detachment and degeneration as tissue inflammation progresses, which can cause early tooth loss [92,93]. Li et al. developed a hydrogel patch for multiplex drug delivery of antibiotic and immunomodulatory cytokines for promoting periodontal tissue regeneration [94]. However, more than just dental treatment is required for current medical development as diagnosis is as important as dental treatment. It is crucial to develop a platform that can achieve the timely treatment and diagnosis of the oral problems at the same time as providing proper dental care. When food residue remains on teeth, a tooth lesion can occur as the oral bacteria metabolize food residues and produce acid on the tooth surface, demineralizing the teeth over time. Liu et al. constructed a hydrogel theranostic dental patch for intraoral sensing and drug delivery to wirelessly monitor the oral microenvironment and cure dental diseases caused by oral microbiome dysbiosis [89]. The dental theranostic patch can achieve on-demand fluoride delivery by electrical stimuli to arrest the lesion at an early stage, effectively promoting tooth remineralization and inhibiting the growth of lesion-related bacteria (Figure 11B). The patch was also used as an electrochemical potentiometric sensor to monitor the changes in the tooth microenvironment, especially the pH change, and as a way to wirelessly transmit data to the equipment in real time. Moreover, the patch employed hydrogel to ensure the wearing safety and comfort, improving the fitting between teeth and the theranostic platform. 

### 3.5. Hydrogel Bioelectronics for Nerve-Related Applications

Conductive electrodes have been used as an interface with the nervous system for neuroscience research, diagnosis, and therapy applications [95,96,97]. However, many implantable neural electrodes might induce an adverse body response as the neural electrodes usually require long-term employment [98,99]. Therefore, electrode mechanics have become a promising alternative for nerve-related applications. Notably, soft electronics with flexibility can further minimize the mechanical mismatch and stiffness of neural electrodes [100]. Hydrogel has been increasingly used in neural electrodes due to its intrinsic softness and significant biocompatibility. Hydrogel can also mimic the extracellular matrix of neural tissue to provide a favorable environment for neural cells to grow, differentiate, and function [101,102].

**Figure 12:**
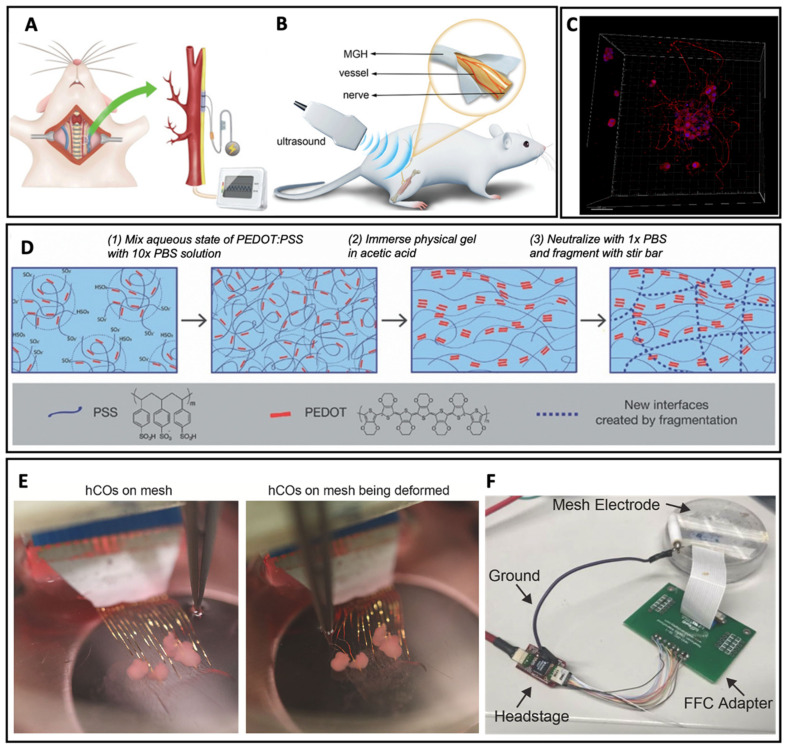
(**A**) Schematic illustration of rat vagus neuromodulation for blood pressure control. (**B**) Schematic illustration of ultrasound imaging of MGH/nerve interfaces. Reproduced with permission from Wiley-VCH, copyright 2022 [103]. (**C**) Top view of neurite outgrowth of PC12 and SCs co-cultures in 10 wt% PVA-SG. Reproduced with permission from Elsevier, copyright 2019 [105]. (**D**) Schematic illustration of the fabrication process of granular conductive hydrogels. Reproduced with permission from Wiley-VCH, copyright 2021 [106]. (**E**) Images of the mesh electrode with organoids and manipulation of metal forceps. (**F**) Images of connection between the mesh electrode and the Stim/Recording controller. Reproduced with permission from Elsevier, copyright 2022 [107].

Zhang and Li et al. constructed a hydrogel-based membrane for interfacing chronically with nerves [103]. The neural interface was formed based on graphene, which was a commonly used neural electrode but with limited ion-accessible electrode surfaces and compromised conductivity. Therefore, to improve the conductivity, a multilayered graphene hydrogel membrane was designed with a continuous open core structure to allow a fast charging rate and high electrical capacitance simultaneously. The constructed graphene hydrogel membrane can be used for vagus neuromodulation by encircling the membrane around the rat cervical vagus nerves, installing the electrical stimulation on the head, and recording the arterial blood pressure (Figure 12A). The blood pressure was used to determine the effect of the implants for neural functions, and the result indicated that the graphene membrane possessed stable performance for neuromodulation and therapeutic potential for chronic neural interfacing. To further explore the mechanical interaction between the multilayered graphene hydrogel membrane and neural tissues, ultrasonography was utilized (Figure 12B): the membrane presented small impacts in the neural blood flow and reduced inflammation, indicating the hydrogel membrane as a promising platform for chronic neuromodulation. Therefore, hydrogel with significant ion-accessible surfaces and distinctive biocompatibility is favorable to employ as bioelectrodes. Bao et al. also designed a hydrogel electrode for localized low-voltage neuromodulation [104]. The electrode was based on electronic conductive hydrogel, and the hydrogel had an electrical conductivity of 47.4 ± 1.2 S cm^−1^. 

Besides the neural interface, hydrogel can also be employed to promote nerve regeneration. Aregueta-Robles et al. engineered a tissue hydrogel to support 3D neural networks, demonstrating a feasible approach for the simultaneous seeding, growth, and differentiation of neural cells within hydrogel [105]. The hydrogel was copolymerized with biomacromolecules to form PVA-SG with nerve tissue-like mechanical properties, which was further used to co-culture Schwann cells (SCs) and neural-like PC12s. As shown in Figure 12C, the hydrogel-based cell cultures can associate with adjacent networks or clusters to form more extensive networks across the scaffold, indicating hydrogel is a promising material for neurite outgrowth towards target tissue. 

Conductive cell scaffolds have been developed to stimulate stem cells to improve cell attachment, promote cell proliferation, and intensify cell differentiation to osteogenic, neurogenic, and cardiac lineages [108]. However, traditional electrical scaffolds are unsatisfied with the leveraged electrical functionalities due to the mismatch between the electronic materials and soft tissues [109,110]. Conductive hydrogel with excellent flexibility and tissue-like mechanical properties is a promising material of cell scaffolds [19,29,111]. George and Bao et al. constructed conductive granular hydrogel for injectable 3D cell scaffolds to provide a platform of manipulating stem cell behavior [106]. The hydrogel scaffold was fabricated easily, as shown in Figure 12D, to form injectable 3D niches with shear-thinning and self-healing abilities, which made it promising in regenerative medicine with electric field manipulation. The PEDOT-based hydrogel can interact with stem cells due to its controllable hydrogel properties and unique cellular biocompatibility.

Compared with traditional electrodes, mesh electrode, as an emerging platform for chronic electrophysiological interfaces with brain tissue, is characterized by the flexible conductive interconnects with insulating encapsulation due to its easy conformation to neural tissue and potential to minimize foreign body interactions [112,113,114,115]. Since the substrate modulus of mesh electrodes can influence cell behavior, and soft stretchable mesh electrodes can minimize mechanical disturbance during cell growth, Bao, Cui, and Pasca et al. developed hydrogel-based mesh bioelectronics for the biointegration and stimulation of human neural organoids [107]. As the hydrogel allowed the mesh electronics to stretch highly with minimal force and without influence on the electrical performance, the hydrogel-based mesh electrode could deform easily in the culture by manipulation with metal forceps (Figure 12E,F), demonstrating significant stability and stretchability. In detail, the stable electrochemical impedance of the hydrogel-based mesh electrode was maintained in buffer solution under 50% compressive and 50% tensile strain (two-way ANOVA, *p* = 0.921 for strain). To further test whether pluripotent stem-cell-derived human cortical organoids (hCO) could be stimulated on the mesh electrode, the mesh organoid was connected to an Intan RHS controller to perform stimulation experiments, which demonstrated that the hydrogel electrode triggered intensity-dependent calcium signals and was promising for monitoring the electrical activity of neuropsychiatric diseases.

## 4. Conclusions and Outlook

With the popularization of 5G intelligent terminals and the improvement in intelligent medicine, multifunctional flexible electronic devices play an irreplaceable role in intelligent medical treatment and motion monitoring. Flexible electronic devices can send physiological electrical signals and motion states to smart terminals. Through artificial intelligence and extensive data analysis, health and motion states can be monitored remotely and in real time. Therefore, preparing materials for flexible electronic devices has become a research hotspot. Traditional electronic devices have problems such as a hard texture, fragility, and a mismatch between their mechanical properties and skin. Flexible electronic devices have excellent tensile properties, electrical conductivity, and similarity to the skin modulus, which can perfectly solve the drawbacks of traditional rigid electronic devices.

Hydrogel bioelectronics have made significant progress in their mechanical properties, biocompatibility, self-healing, and temperature sensitivity. However, the development of conductive hydrogels still faces the following challenges: (i) Maintenance of a stable conductivity in the working state. (ii) Maintenance of long-term stability in the face of sudden changes in the environment. (iii) The contact between hydrogel electrodes and biological tissue. If it can avoid causing damage to biological tissues or causing inflammatory reactions, the preparation has excellent mechanical properties. Hydrogel bioelectronic devices with good tissue adhesion, injectability, and biocompatibility are crucial for developing flexible wearable electronic devices.

## Figures and Tables

**Table 1:** Comparison of the tensile strength, conductivity, and gauge factor between conductive hydrogels and metal oxide semiconductors, according to the literature.

Type of Flexible Materials	Composition	Tensile Strength	Conductivity	Gauge Factor	Young’s Modulus	Ref.
Hydrogel	PAA/PANI	1–2 MPa	12 S/m	GF = 11.6, 0–100% GF = 4.7, 100–400%	/	[32]
	PVA/PPy	0.35–0.46 MPa	2.1–5.1 S/m	GF = 3.19, 0–250% GF = 6.77, 250–500%	112.7 kPa	[33]
	P(HEAA-co-SBAA)/PEDOT:PSS	0.15–0.5 MPa	0.006 S/m	GF = 2, ~5000%	/	[34]
	PPy@cellulose	0.175 MPa	0.5–4.5 S/m	GF = 8.4, 300%	/	[35]
Metal oxide semiconductors	Indium–gallium–zinc oxide (IGZO)	110 GPa	0.004 S cm^−1^	/	110 GPa	[36]
	IGZO:polytetrafluoroethylene (PTFE) films	0.3 GPa	/	/	104 GPa	[37]

## Data Availability

Not applicable.
